# Reconsidering evidence-based management of endometriosis

**DOI:** 10.52054/FVVO.14.3.033

**Published:** 2022-09-30

**Authors:** P.R. Koninckx, A Ussia, S Alsuwaidi, B Amro, J Keckstein, L Adamyan, J Donnez, M.C. Martin, A Wattiez

**Affiliations:** Latifa Hospital, Dpt OBGYN, Dubai, United Arab Emirates; Prof Emeritus OBGYN KULeuven Belgium, University of Oxford, and Hon Consultant UK, University Cattolica, Roma, Italy and Moscow State Univ, Russia; Consultant Università Cattolica, Dpt OBGYN Roma Italy; Endometriosis Centre, County Hospital Villach, Austria and University Ulm, Dpt OBGYN, Ulm, Germany; Department of Operative Gynecology, Federal State Budget Institution V. I. Kulakov Research Centre for Obstetrics, Gynecology, and Perinatology, Ministry of Health of the Russian Federation, Moscow, Russia; and Department of Reproductive Medicine and Surgery, Moscow State University of Medicine and Dentistry, Moscow, Russia; Prof emeritus Catholic University of Louvain, Dpt OBGYN, Brussels, Belgium; Prof Emeritus, Dpt OBGYN, University of Tennessee Health Science Centre, Memphis Tennessee, USA; Institutional Review Board, Virginia Commonwealth University, Richmond, Virginia, USA; Prof Department of obstetrics and gynaecology, University of Strasbourg, France

**Keywords:** evidence, statistical inference, Bayesian, endometriosis, evidence-based medicine

## Abstract

**Background:**

Without an adequate animal model permitting experiments the pathophysiology of endometriosis remains unclear and without a non-invasive diagnosis, information is limited to symptomatic women. Lesions are macroscopically and biochemically variable. Hormonal medical therapy cannot be blinded when recognised by the patient and the evidence of extensive surgery is limited because of the combination of low numbers of interventions of variable difficulty with variable surgical skills. Experience is spread among specialists in imaging, medical therapy, infertility, pain and surgery. In addition, the limitations of traditional statistics and P-values to interpret results and the complementarity with Bayesian inference should be realised.

**Objectives:**

To review and discuss evidence in endometriosis management

**Materials and Methods:**

A PubMed search for blinded randomised controlled trials in endometriosis.

**Results:**

Good-quality evidence is limited in endometriosis.

**Conclusions:**

Clinical experience remains undervalued especially for surgery.

**What is new?:**

Evidence-based medicine should integrate traditional statistical analysis and the limitations of P-values, with the complementary Bayesian inference which is predictive and sequential and more like clinical medicine. Since clinical experience is important for grading evidence, specific experience in the different disciplines of endometriosis should be used to judge trial designs and results. Finally, clinical medicine can be considered as a series of experiments controlled by the outcome. Therefore, the clinical opinion of many has more value than a personal opinion.

## Introduction

Medicine is based on observations and experience and statistical analysis to help with the interpretation of observations. For more than a century classical statistical methods or the frequentist approach (Fisher 1925; Neyman and Pearson, 1928) used significance levels, power and P-values to grasp in one value the probability that an observed effect, can be attributed to chance (null hypothesis), taking into account its distribution and power. P-values measure the extremeness of a result given the null hypothesis but do not evaluate whether the hypothesis is true (Lesaffre and Lawson, 2012). This mistake is often made in medicine and is known as the P-value fallacy (Goodman, 1999). Recently the American statistical association published a statement that “P-values do not measure the probability that the studied hypothesis is true or the probability that the data were produced by random chance alone and that they are not a good measure of evidence regarding a model or hypothesis” (Wasserstein and Lazar, 2016). Statistical reporting, therefore, is changing in gynaecological journals (Hardwicke and Goodman, 2020) such as the Journal of Minimally Invasive Gynecology (JMIG) and the British Journal of Obstetrics and Gynaecology (BJOG) (Price et al., 2020; Wilson and Falcone, 2020).

Evidence-based medicine (EBM) was developed to integrate research data corrected for biases into clinical medicine. EBM, initiated in the 1990s when calculators permitted more complex analyses and meta-analyses, embraced P-values for evidence resulting in a pyramid of evidence (Djulbegovic and Guyatt, 2017) with the randomised controlled trials (RCT) (Lawrence and Force, 1989), and later meta- analyses (CEBM, 2009 ) and systematic reviews on top. However, the translation of results in grades of evidence and the integration of research data in clinical medicine and guidelines proved to be difficult (Murad et al., 2016a). Today, this difficulty could be seen as the unconscious conflict between the inappropriate use of P-values as ‘evidence’ for the initial hypothesis, and clinical medicine using a rather Bayesian approach for diagnosis and therapy.

An EBM approach to endometriosis needs specific considerations. The absence of adequate animal models limits experiments, and the pathophysiology remains debated and it is still unclear whether endometriosis is one or several diseases. Without an adequate non-invasive diagnosis, epidemiology is poorly understood. RCTs for medical therapy are hampered by the absence of blinding when the patient recognises therapy (e.g., when affecting menstruation). Extensive surgery is too variable for the available number of interventions to permit RCTs.

Considering our recently changing understanding of statistical inference, we will review the EBM approach to endometriosis and highlight clinical experience and judgment in the hierarchy of evidence.

## Materials and Methods

A PubMed search “endometriosis AND “double- blind” AND (recurrences OR pain OR fertility) AND (surgery OR medical OR surgical)” yielded 101 results. These were hand searched to exclude so-called placebo-blinded trials when blinding was inadequate e.g., when active treatment was readily recognised by the patient or the gynaecologist by changes in menstruation or menopausal symptoms or vaginal atrophy. When recognition was not clear and when placebo effects could not be judged, trials comparing 2 hormonal therapies such as dienogest and GnRH agonists after surgery (Ceccaroni et al., 2021) were excluded. In addition, 1 trial dealing with acupuncture and 7 trials describing vitamin D or fatty acid treatment and 1 trial with anti-TNFa and 1 with pentoxifylline treatment was eliminated. After this, only 2 trials of laser vaporisation of superficial endometriosis and 4 trials comparing excision with coagulation or ablation of superficial endometriosis, remained. Prism flow sheets are no included, since not adequately blinded RCT describing hormonal medical therapy of endometriosis and no RCT reporting surgery of severe endometriosis were not found.

### Classical and Bayesian statistical inference are complimentary

Without discussing classical and Bayesian statistics in detail, the clinician should grasp the differences and the complementarity. Traditional statistics evaluate the probability that the results of an experiment could be obtained by chance alone, without considering previous knowledge or other experiments. Traditional statistics therefore can only refute but not confirm a hypothesis. Judgement of the validity of a hypothesis rather requires Bayesian reasoning, needing a prior probability and the Bayesian factor indicating how experimental results change that probability (Goodman, 2001; Sellke et al., 2001). The relationship between traditional and Bayesian statistics can be illustrated as follows. If we do not know whether something is true or not the (prior) probability is 50%; an experiment demonstrating an effect with a P-value of 0.05 or 0.01 increases this probability to 71% or 89% respectively. This also emphasises the uncertainty, since the initial hypothesis remains wrong in 29% and 11%, respectively (Nuzzo, 2014). The probability that a hypothesis is correct moreover varies with many other factors besides P-values (Goodman and Greenland, 2007).

Bayesian reasoning is rather sequential and the probability that a hypothesis is true, based on previous knowledge, is updated with the new information. The weather forecast, which is more accurate for tomorrow than for the following days, is daily updated with new information. This is more like medical thinking where a diagnosis is progressively refined, and a therapy updated with new information.

This improved understanding of statistics, as discussed over the last decades, explains that observations in medicine are often poorly reproducible (Boos and Stefanski, 2011; Colquhoun, 2017) and that the conclusion that many research findings in medicine are wrong (Goodman and Greenland, 2007) and often "an accurate measure of the prevailing biases" (Ioannidis, 2005). However, classical and Bayesian statistics are complementary. The former is more suited to evaluate the multiple interactions in a large dataset (multivariate analysis), the former being more suited to predict the future or the accuracy of a hypothesis.

### Hierarchy of evidence in EBM

‘Evidence-based medicine is the conscientious, explicit, and judicious use of current best evidence in making decisions about the care of individual patients’ (Sackett et al., 1996). By formalising RCTs, reporting, meta-analyses, systematic reviews, and the prevention of selection or observation biases, data became more reliable, improving the quality of evidence (Djulbegovic and Guyatt, 2017). However, the integration of EBM into clinical medicine was difficult (Djulbegovic and Guyatt, 2017). Today we understand that this was to some extent a consequence of considering P-values erroneously as a confirmation of a hypothesis instead of changing the probability. Although more comparable to clinical decision- making for diagnosis and therapy, Bayesian inference (Lesaffre and Lawson, 2012) is still poorly incorporated in EBM.

### Considerations of traditional analysis of treatment and diagnosis

**Figure 1:**
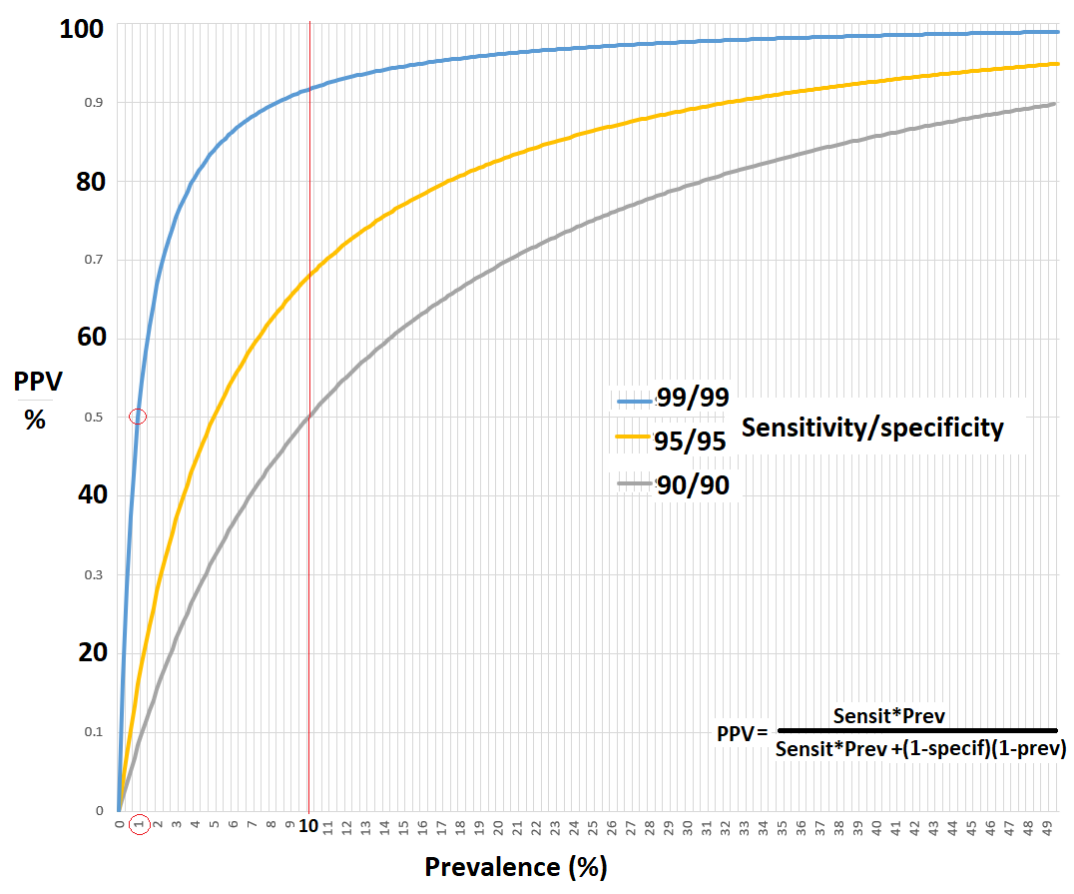
Positive predictive values were calculated for tests with sensitivities and specificities of 90% to 99% when prevalence varies from 1% to 50%.

Although well-known, clinicians risk forgetting that traditional statistical analyses require a homogeneous population and that they are not suited to detect subgroups (Koninckx et al., 2020c) with different behaviour, or for rare events. The latter requires (often prohibitively) large groups to be evaluated. A historic example is that it took many years to realise that chloramphenicol, an excellent antibiotic, had a 1/10.000 risk of aplastic anaemia (Polin and Plaut, 1977). Another problem is the publication bias (Lin and Chu, 2018; DeVito and Goldacre, 2019) when small, clinically irrelevant differences reach ‘significance’ because of large groups (since P-values improve with the square root of the number of observations). Diagnostic tests are used to estimate the probability that a patient has or does not have a disease, i.e., the positive (PPV) or negative predictive value (NPV). However, the accuracy of prediction decreases sharply when the prevalence of a disease is low, especially when lower than 5 or 1%. This is shown in Figure 1 using the Bayesian formula (Lesaffre and Lawson, 2012) to calculate this relationship, illustrating that a test with 99% sensitivity and 99% specificity for a disease with a 1% prevalence, has a predictive value of only 50%, the number of false positives and true positives being similar. Unfortunately, reports describing the accuracy of tests rarely specify prevalence, which moreover increase because of a referral bias. Therefore, the predictive values for rare diseases will be higher in tertiary referral centres (Drossman et al., 1997) than in routine clinical practice. More difficult to calculate, is the combined diagnostic accuracy of several tests (Pepe, 2000). This is illustrated by a recent Cochrane review, suggesting using tests sequentially, beginning with the test with the highest sensitivity, and then re-testing the negative group (Nisenblat et al. 2016). Although the added value of a second test and the combined accuracy of tests can be calculated with a Bayesian approach (Broemeling, 2011) as illustrated recently for endometriosis (Chen and Hwang, 2019; Chen et al., 2019), this is still rarely used.

### Clinical judgment, experience, and artificial intelligence

**Figure 2:**
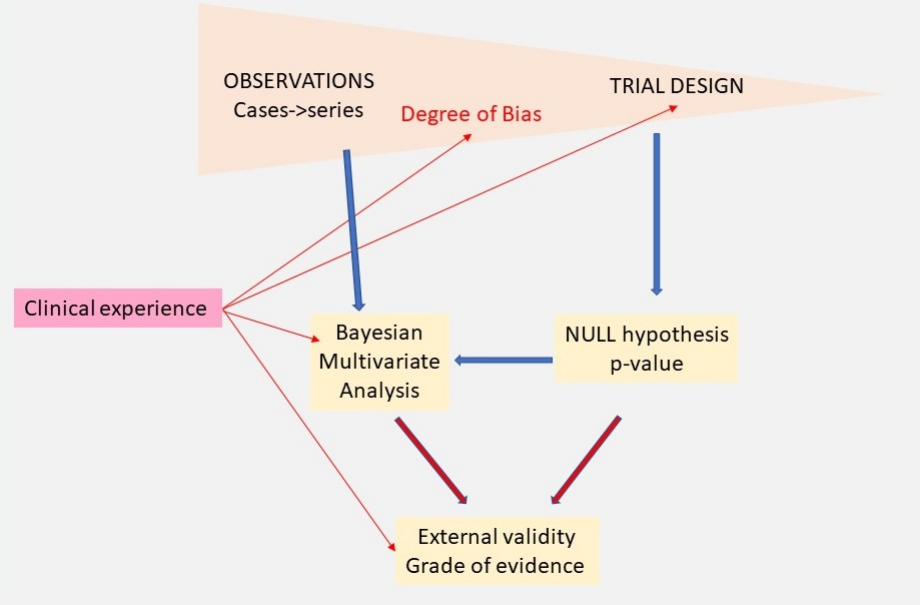
Evidence in medicine starts with observations and trials, the latter having less risk of bias. Traditional statistics test the Null hypotheses resulting in P-values and are more suited for multivariate analysis. Bayesian analysis is more sequential and better suited to judge the hypothesis of the trial. Clinical experience is important for all aspects and judges the risk of bias, provides the prior information to start trials, orient analysis and evaluates external validity and grades of evidence. Considering the importance of clinical experience, the variability of experience by the sub-disciplines in endometriosis needs to be formally addressed in diagnosis and therapy.

Clinical judgment is important although difficult to standardise. Some data cannot be compared, such as the efficacy of treatment versus the severity or incidence of side effects. In surgery, both are moreover linked since surgeon dependent. When blinding is not possible, efficacy and placebo effects cannot be separated. Also, the judgment of imprecision, inconsistency, publication bias and external validity, is difficult. We should realise that the same holds for the grades of evidence used in EBM (Murad et al., 2014; Murad et al., 2016a). More subtle is how the criteria used to judge the quality of RCTs (Mahmood et al., 2021) influence the conclusions of meta-analyses up to becoming misleading (Ioannidis, 2016). Although clinical judgment is difficult to define, its value was illustrated by the observation that the formal evaluation of biases in diagnostic accuracy by Quadas tools (Whiting et al., 2011; Yang et al., 2021) is not much superior to clinical judgment. Clinical experience integrates knowledge with experience in the entire population, including heredity, age, antecedents, and rare events. Clinical experience thus is much larger than evidence derived from trials since many trials are not performed when clinical consequences are minimal, or when prevalence is low as in multimorbidity. Many individual and local preferences moreover were implemented following rare events, accidents or near accidents, which become forgotten years later. Therefore, we should be prudent when changing habits because of lack of evidence. Clinical decision-making (You and Krumholz, 2022) is complex. Considering the age, antecedents, symptoms, clinical exam, blood tests and imaging, the clinician considers a series of potential diagnoses, that range from most likely to rare. The integration of all these variables into a PPV and NPV for each diagnosis considered, including the risk when making a mistake, is a progressive complex experience-based, artificial intelligence-like (Letterie, 2021) process. Finally, it should be realised that clinical experience precedes and guides RCTs (Figure 2). They are performed to confirm an observation, or not performed when the superiority of a treatment or an intervention seems repetitively observed, without exceptions, or when the expected effect is so little that the result will be clinically irrelevant.

Emotional intelligence is rarely considered since difficult to define. However, the interaction of the clinician and the patient through body language and expectations of the patient influences diagnosis and therapy, and similar data can be interpreted differently by clinicians with comparable experience.

## Conclusion

These considerations explain the difficulties of the clinical integration of EBM and the ongoing discussions on the hierarchy of evidence (Murad et al. 2016b). Besides the interpretation of data and the fallacy of p-values, grades of evidence were introduced to consider all available evidence including observational series and case reports, emphasising the importance of clinical judgment and experience.

In addition, we did not discuss other limitations of EBM and the hierarchy of evidence, such as publication bias (Vercellini, 2014; DeVito and Goldacre 2019), procedural aspects such as financial bias in funding (Howick, 2019) and drug research (Klemperer, 2010), the epistemological discussion to distinguish justified belief from opinions (Gaeta and Gentile, 2016) and the importance of medicolegal aspects as described in the recent introduction of NUTS (Number of Unnecessary Tests to avoid one Suit) statistics (Allen et al., 2021).

## EBM and Endometriosis

### Specific problems of endometriosis

Endometriosis is a frequent disease causing pain and infertility and is the most frequent reason for surgery in women (Kempers et al., 1960). Given the likely association with adenomyosis and bleeding disorders (Koninckx et al., 2018), endometriosis can be considered for almost any complaint in gynaecology. Without an adequate animal model permitting experimentation and without a non- invasive diagnosis, the pathophysiology, the natural history, and the epidemiology (Koninckx et al., 2021a) are poorly understood. Data on endometriosis are scanty in some age groups such as adolescence and overall limited to symptomatic women except small series (Moen and Stokstad, 2002). The latter is illustrated by the recent suggestion by statisticians to redefine endometriosis as ‘symptomatic’, thus facilitating data handling but eliminating those who did not undergo a laparoscopy (Goodman and Franasiak, 2018). Even for cystic ovarian endometriosis the accuracy of imaging seldom exceeds 90% while it remains difficult to exclude ovarian cancer, especially in older women (Van Holsbeke et al., 2009).

### Good-quality data are limited

Most laparoscopies are performed in symptomatic women. In individual women, it is difficult to ascertain whether endometriosis and pain or infertility are causally related. Only half of the superficial lesions are painful (Demco, 2000) and there are many other causes of pelvic pain or infertility. Medical therapies have an important placebo effect (Koninckx et al., 2008), and blinding is an illusion when the patient recognises active therapy by affecting menstruation or menopausal symptoms. Although most trials require a proven endometriosis diagnosis for inclusion, it is unclear which endometriosis they have after laparoscopy with surgical destruction. Judgment of a trial can vary over time, as illustrated by the not-blinded ENDOCAN trial (Marcoux et al., 1997) showing improvement in fertility following surgery. A Cochrane meta-analysis was subsequently withdrawn (Jacobson et al., 2014). For cystic ovarian endometriosis the results of surgery, ovarian damage and recurrence rates are surgeon- dependent (Muzii and Miller, 2011). Deep endometriosis is highly variable, and surgery is technically difficult and complication prone. Because of the variable skills of the surgeon, and the low number of interventions, RCTs are not realistic. If performed nevertheless, unexpected results risk being criticised as occurred recently in the LACC trial demonstrating higher recurrence rates after laparoscopic surgery for cervical cancer (Ramirez et al., 2018).

### Clinical judgment varies with subspecialties

The clinical judgment of endometriosis is complicated by different sub-specialists. Clinical experience is bound to vary between radiologists performing magnetic resonance imaging (MRI), gynaecologists specialising in ultrasonography or endocrinology or medical therapy or surgery, and abdominal surgeons with little expertise in other aspects of gynaecology. An additional difficulty is the degree of commercialisation and industrialisation (Perrotta and Geampana, 2021), especially in infertility and medical therapy.

### Clinical judgment varies with our perception of pathophysiology

Management should be based on evidence, but our clinical judgement might vary with our understanding of pathophysiology. The implantation theory (Sampson, 1921; Sampson, 1927) defined endometriosis as ‘endometrial glands and stroma outside the uterus’ and thus as one disease, which became clinically considered progressive and recurrent. According to the genetic-epigenetic (G-E), theory endometriosis starts developing after a cumulative but variable series of cellular incidents (Koninckx et al., 2020a). This is consistent with endometriosis lesions being clonal and variable as observed for aromatase activity and progesterone resistance (Bulun et al., 2019), and for the response to medical therapy (Becker et al., 2017; Vercellini et al., 2021). If lesions are different, traditional statistical analysis with means and standard deviations is inadequate (Koninckx et al., 2020c). Since the risk of G-E incidents increases by the oxidative stress of retrograde menstruation or the peritoneal microbiome, it is logical that susceptible women have an increased risk after puberty, and the remaining group will have a progressively lower risk (Koninckx et al., 2021c). Thus, age becomes an important factor in epidemiology. Pelvic endometriosis lesions grow in the peritoneal cavity which is endocrinologically and immunologically a specific microenvironment. The growth of endometrial lesions is self-limiting (Koninckx et al., 2021c) probably as a consequence of fibrosis and inflammation secondary to the immunologic reaction. This is consistent with the clinical observation that most deep endometriosis lesions that are followed clinically since symptoms were insufficient for surgery, do not grow. Viewed as a G-E-driven disease, recurrences might become preventable by decreasing oxidative stress. This is consistent with the lower recurrence rate of cystic ovarian endometriosis when taking oral contraception. Although not demonstrated yet, we might consider prevention by preventing ascending infections, or by changing the peritoneal microbiome by food intake and exercise (Langweiler, 2020). This is consistent with the observations that the risk of developing endometriosis seems lower when taking food rich in antioxidant as omega3, Vit E, Vit C, and citrus (Harris et al., 2018; Afrin et al. 2021). It is too early to fully understand the effect of vitamins on inflammation and immune response in endometriosis (Halpern et al., 2015). New concepts of pathophysiology should be considered for future trials. This could apply more specifically to endometriosis in adolescence, to the prevention of endometriosis, and to interpret results of endometriosis if heterogeneous and more than one disease.

### Non-biomedical health systems

A growing number of reports document the management of endometriosis with complementary therapies (Porpora et al., 2013; Mira et al., 2018; Della Corte et al., 2020), acupuncture, food intake (Nirgianakis et al., 2021) and exercise (Langweiler, 2020), and more recently traditional Chinese medicine (Peng et al., 2021). These reports are difficult to interpret since the indications and results of these treatments poorly fit EBM standards. However, indirect, and circumstantial evidence is too strong to ignore these treatments altogether. We are at the crossroads of understanding the role of food intake and exercise on the peritoneal (Koninckx et al., 2019) and the intestinal microbiome. Both might influence endometriosis onset and growth either directly or through immunology and oxidative stress.

## Conclusion

In endometriosis high-quality evidence is very limited and the clinical judgment varies with experience that is different for the subspecialties involved. These differences in experience should be addressed since experience affects the grading of evidence and the recognition of bias. It seems logical that the ranking of evidence for diagnosis, medical therapy and surgery should be performed by those with experience in that subspecialty.

Surgery for severe endometriosis requires specific comments. Data are limited to observational series with referral biases and important differences in technique. However, the surgeons with extensive experience are a small group, who know each other’s surgery and who meet and discuss several times a year and progressively adapt their surgery (Donnez and Donnez, 2021). Therefore, the elements on which this group agrees because of a similar experience have more value than an opinion. Each intervention can be seen as an experiment testing management and measured by outcome. Hopefully, statisticians will help to formalise this collective experience and outcome-based observations into evidence.

## Discussion

The principles of EBM (Sackett et al., 1996) are clear, but the hierarchy of the evidence is struggling with a poorly transparent clinical judgment, which for endometriosis might vary with the experience of the subspecialists. It seems logical to match the judgment of evidence with the extent and type of experience in each subspecialty.

Evidence needs to be translated into guidelines. This requires the input of all stakeholders. Also, the interpretation might vary over time with changing understanding of pathophysiology. Whether endometriosis is considered as one or several G-E different diseases will help to understand that some 50% of typical lesions are not painful and that response to medical therapy is absent or inadequate in 10% to 40% respectively (Donnez and Dolmans, 2021; Vercellini et al., 2021). Today it seems logical that superficial, cystic ovarian and deep endometriosis are reported separately since likely different entities (Donnez et al., 1996).

Clinicians have been educated with significances and P-values, and risk having misused them to confirm a hypothesis (Nuzzo, 2014). A full discussion of statistical inference being beyond this manuscript, the differences between traditional (P-values) and Bayesian (probabilities) statistics can be illustrated as follows. A 60% probability of rain is different from a non-significant P-value that it is going to rain. Although important, it will take time to incorporate a Bayesian approach (Lesaffre and Lawson, 2012) and to acknowledge the similarities with clinical medicine. First, consider the progressive approach of clinical medicine and Bayesian inference: a clinician seeing a woman of this age, with these antecedents and these symptoms, considers many differential diagnoses which are refined into a workable probability by additional exams and tests. This finally results in a conclusion or treatment considering the consequences of mistakes and complications. In addition, we have to prevent the gap between statistical inference and clinical understanding from becoming wider, as illustrated by a recent diagnostic test of endometriosis using a ‘penalised regression model and machine learning with random forest’ (Moustafa et al., 2020). This risks not being readily understood by most clinicians.

The strength of evidence needs to be re- evaluated for hormonal medical therapy of endometriosis. First, we need to acknowledge that adequate blinding cannot be done when the patient recognises active therapy. In addition, we need to incorporate the high peritoneal fluid concentrations and progesterone resistance (Donnez and Dolmans, 2021; Koninckx et al., 2022) and that endometriosis lesions are a heterogeneous group (Koninckx et al., 2020c; Donnez, 2021) with variable growth even during medical therapy (Vercellini et al., 2021) or after menopause (de Almeida Asencio et al., 2019). We also should avoid vague clinical terms such as “adequate pain relief”, or ‘women with proven endometriosis’. Notwithstanding these considerations, the clinical treatment of superficial endometriosis could be summarised as follows. Women with proven or suspected endometriosis and pain deserve a trial with medical therapy, but the eventual growth of lesions during therapy should be monitored and if pain relief is inadequate, other options should be considered.

Judgment of surgery remains difficult. Quality is poorly defined, the severity of endometriosis is variable, and cystic and deep endometriosis is technically difficult and complication-prone surgery with oocyte damage, sexual problems and bladder, ureteral and bowel complications (Koninckx et al., 2021b). Randomisation is unrealistic and often unethical when surgeons are not equally trained in the techniques to be compared. Since this is unlikely to change, it seems important to use a Bayesian approach to establish the value of the collective experience of surgeons in technique, results and complications and the importance of granular intra-operative details (Kanters et al., 2018). This is not contradicted by the decision of doing a bowel resection or a conservative excision or a discoid excision being based to a large extent on personal preferences (Koninckx et al., 2020b) since results and complications vary with surgical skills and experience. To convert this collective opinion based on repetitive surgical interventions with the outcome as measurement, into a degree of evidence will be a methodological and statistical challenge.

In conclusion, an EBM approach to endometriosis faces specific challenges. The diagnosis is limited to those undergoing laparoscopy and this decision is based on a variable mixture of clinical exams and symptoms and imaging. The accuracies of imaging such as ultrasound or MRI are well described (Guerriero et al., 2021a; Guerriero et al., 2021b), but the predictive values vary with the prevalence, and their importance in clinical decision-making varies from little (Koninckx et al., 2021b; Koninckx et al., 2021c) to very much (Malzoni et al., 2020). We need to incorporate that the recognition of subtle and deep endometriosis (Taylor et al., 2018) is variable. Medical therapy needs re-appraisal and for extensive surgery the value of the collective judgement of surgeons needs evaluation. This complexity will need better integration of traditional and Bayesian statistical analysis and inference to understand which exams and therapies improve outcomes (Bernstein and Wang, 2021).
